# Global determinants of yield variability under sustainable farming approaches across climate, soil, and topography. A meta-analysis

**DOI:** 10.1007/s13593-026-01133-7

**Published:** 2026-07-22

**Authors:** Kpade O. L. Hounkpatin, Johannes Piipponen, Emanuela De Giorgi, Mika Jalava, Jeroen Poelert, Matti Kummu

**Affiliations:** 1 Water and Development Research Group, Aalto University, Espoo, Finland; 2 Department of Environment, Land and Infrastructure Engineering, Politecnico di Torino, Torino, Italy

**Keywords:** Sustainable farming approaches, Soil properties, Topography, Climate variables

## Abstract

Enhancing ecosystem services without compromising crop productivity is a central challenge for sustainable agriculture, yet biophysical drivers of yield variability across farming approaches remain poorly resolved. Here, we provide the first comparative global assessment of how climate, soil, and topographic conditions shape yield responses to agroforestry, cover cropping, no-tillage, and organic farming by synthesizing global meta-analytic datasets and linking field comparisons to a common framework of biophysical moderators. Across all sustainable farming approaches, there was no significant overall yield difference relative to conventional management (1.1%; 95% confidence interval –0.7 to 3.0%), but outcomes diverged across biophysical contexts. While agroforestry and cover cropping showed statistically significant positive yield effects under specific aridity conditions, no-tillage was consistently associated with statistically significant yield reductions in wetter environments, and organic farming showed no significant overall effect. Responses across soil and topographic gradients were context-dependent, with significant effects observed for specific soil types and elevated or sloping landscapes, whereas most other patterns were not consistently statistically significant. These findings demonstrate that environmental heterogeneity governs yield responses to sustainable farming approaches and support context-specific implementation strategies to improve agricultural sustainability and resilience.

**Supplementary information:**

The online version contains supplementary material available at 10.1007/s13593-026-01133-7.

## Introduction

More than 70% of the Earth’s land, originally covered by forests and natural ecosystems, has been converted for human use, with agriculture alone accounting for approximately 40% of the global land area (UNCCD 2022). However, feeding the world has come with a heavy toll on the planet’s soils, with nearly one-third of agricultural land now showing moderate to severe degradation, threatening long-term food security (IPBES 2019; Smith et al. 2024). Meanwhile, it is projected that food production will have to increase in the future to satisfy both the need of the global growing population and the increase in per capita demand (Bodirsky et al. 2015). In this context, sustainable pathways that contribute to land restoration, biodiversity protection, and GHG mitigation are increasingly emphasized.

In response to these mounting pressures on land and food systems, a broad suite of sustainable farming approaches (SFAs) is increasingly promoted to enhance food production while restoring soil health, reducing reliance on external inputs, and minimizing environmental degradation (Ponisio et al. 2015; Rockstrom et al. 2017). Within this context, a range of approaches is commonly implemented, including agroforestry (AF), cover cropping (CC), reduced or no-tillage (NT), and organic farming (OF) (Khangura et al. 2023; Rehberger et al. 2023). Studies report multiple potential benefits of these SFAs, such as increases in soil organic carbon (SOC), improvements in soil water infiltration and storage, and reductions in greenhouse gas emissions (Haddaway et al. 2017; Khangura et al. 2023). Despite promising environmental outcomes, the effects of these SFAs on crop yields remain complex and context dependent.

Yield outcomes under different SFAs have indeed shown mixed results. Some studies have shown that implementation of SFAs could potentially result in increasing yields (Ren et al. 2023; Su et al. 2021), while others reported neutral or declining trends (Giller et al. 2021). Yields increased in arid regions but declined in tropical regions with maize-based systems (Pittelkow et al. 2015). A global meta-analysis based on 740 paired measurements from 90 peer-reviewed articles showed that NT increased barley yield by 49% especially in dry climates (Huang et al. 2018). In a drought period, about 60% higher maize yields were observed under NT management compared to conventional management (CM) (Al-Kaisi and Lal 2020). However, contrary trends are also reported, with the application of crop rotation, residue management, and no-tillage having no effect on yield stability relative to CM (Knapp and van der Heijden 2018). The same study showed that OF had a 15% lower yield compared to CM.

A similar pattern of context-dependent results is seen with other SFAs like AF and CC. Under AF management, findings show that crop yields either increased by 7–16%, especially in subtropical and tropical zones (Baier et al. 2023), or decreased by 2.6% in European areas depending on the density and age of the trees (Ivezic et al. 2021). While about a 14% yield increase was reported under CC, particularly in coarse-textured soils and dryland areas where leguminous cover crops were used (Peng et al. 2024), yield reductions of about 3% were observed, especially for cash crops in temperate soils (Clark et al. 1997; Thorup-Kristensen et al. 2003). About a 10% decrease in wheat yields was observed following cover crop use (Nielsen et al. 2016). In contexts where there is no significant increase or decrease, some studies reported that yields could be sustained over a longer period under SFAs, especially for degraded soils (Lal 2020). The discrepancies of yield outcomes under different SFAs have thus shown that various factors interplay to determine the magnitude and direction of crop yields for farmers.

Crop yields are influenced by a combination of soil, climate, topography, and management practices, all of which play a major role in crop growth and food production (Baker and Capel 2011; Kuradusenge et al. 2023; Wang et al. 2023; Grigorieva et al. 2023). Soil properties determine the local environment for crop growth by affecting soil aeration, nutrient cycling, and root growth (Sainju and Alasinrin 2020; Sainju et al. 2022). For instance, soil properties such as pH, organic carbon content, nutrient levels (e.g., nitrogen, phosphorus), texture, and bulk density directly affect soil water-holding capacity, root growth, and nutrient availability to plants (Mattila and Hakkinen 2025; Oliveira et al. 2024; Strock et al. 2022). Soils with greater soil organic matter levels can better support high yields by retaining moisture and supplying nutrients (Oldfield et al. 2019).

As climate variables, temperature and precipitation are closely associated with crop growth and crop yield and affect soil moisture status, which in turn determines whether water might be a limiting factor in crop phenological development. For example, extreme heat and drought stress can dramatically reduce yields, as evidenced by rising temperatures and more frequent droughts already depressing crop production in many regions (Zhao et al. 2017; Myers et al. 2017). Hence, sufficient rainfall or irrigation, and favorable temperatures during the growing season, is critical for realizing potential yields.

Topographic attributes interact with weather to affect soil temperature and moisture (Leuthold et al. 2022). The landscape position such as elevation and slope gradient influences erosion rates, drainage, and microclimates within a field. Steep or elevated fields may lose topsoil and water to runoff, whereas lower or flatter areas can accumulate moisture but risk waterlogging (Sun et al. 2021; Zhao et al. 2022). Consequently, water stress is more likely to occur in upslope positions with lower and more variable yields compared to lower slope positions (Martinez-Feria and Basso 2020; Leuthold et al. 2021; Basso et al. 2019). Even small changes in slope or aspect create different microclimatic conditions (such as cooler hollows or warmer south-facing slopes) that can affect crop growth (Ewunetu et al. 2025).

Integrating these environmental factors is crucial for developing tailored, sustainable agricultural systems that optimize crop productivity and environmental benefits. However, field experiments and even many meta-analyses often do not report detailed soil metrics, such as bulk density, soil organic carbon, nutrient availability (e.g., phosphorus levels), pH, and texture. They also frequently fail to capture important site characteristics like high elevation and slope, as well as climate indices such as growing degree-days and seasonal moisture levels. Additionally, previous global comparisons of sustainable farming techniques have usually examined one practice at a time (e.g., only no-tillage vs. conventional tillage, or only chemical vs. organic inputs) (de la Cruz et al. 2023; Felix et al. 2018). Such studies rarely attempt to compare the relative effectiveness of multiple sustainable land use practices across different environments and crop types simultaneously. This fragmentary approach leaves a significant knowledge gap in relation to how and why yield responses to different SFAs vary under diverse soil and climate conditions. There remains, therefore, a clear need for a broader understanding of how environmental factors influence crop yield responses under different SFAs.

To address these limitations, recent advances in remote sensing, geospatial modelling, and digital soil mapping provide new opportunities to resolve missing or incomplete data. Global earth datasets can provide information on climate, soil properties, and topography. For example, climate indicators can be sourced from different platforms at a global scale such as CHELSA (Karger et al. 2017), CHIRPS (Funk et al. 2015), and the aridity index (Ahvo et al. 2023). For soil properties, the SoilGrids database offers gridded global maps of soil attributes such as organic carbon content, texture, and pH at multiple depths, derived from thousands of soil profiles and environmental covariates (Poggio et al. 2021). Meanwhile, the SRTM digital elevation model (at ~30 m resolution) and similar terrain datasets capture variations in elevation and slope. Consequently, all these data provide information on environmental conditions and variables related to soil properties, climate, topography, which in turn are potential factors affecting crop yield (Vance et al. 2024; Dash 2024; Kganyago et al. 2024). By extracting these variables for the locations of field experiments, it is possible to characterize each site’s broad environmental context. A limitation of this approach is that such variables should ideally be recorded in the field to ensure accuracy and precision mapping to a particular context. Nevertheless, leveraging global datasets enables large-scale assessments of factors associated with crop yields, allowing us to assess broader patterns where data are limited.

**Fig. 1:**
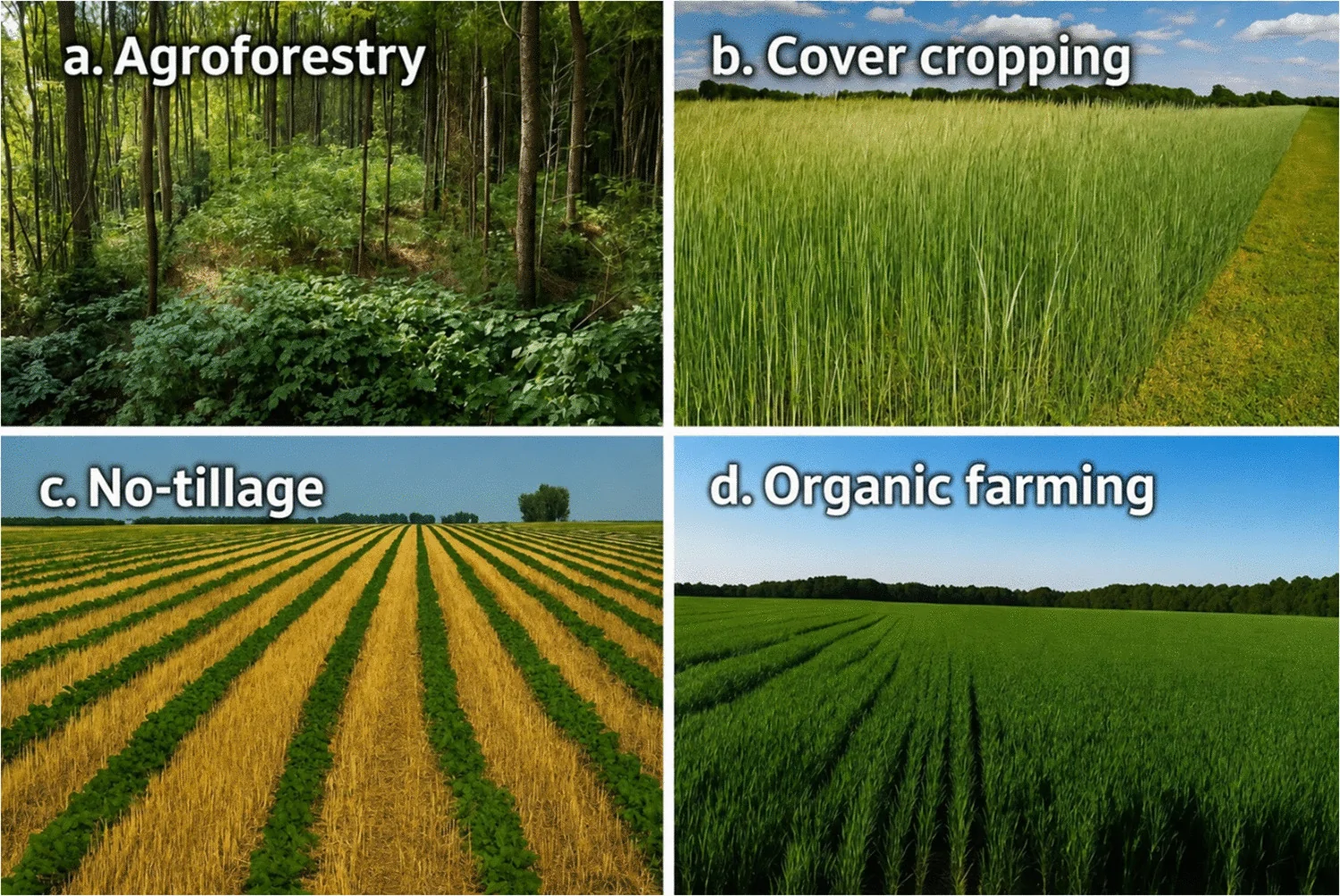
Examples of major sustainable farming approaches evaluated in this study: **a** agroforestry, **b** cover cropping, **c** no-tillage, and **d** organic farming. These photographs illustrate the diversity of management systems that influence soil structure, water retention, and crop productivity across contrasting environments. Photo credits: USDA Agricultural Research Service and USDA Natural Resources Conservation Service (public domain).

In our study, we leverage this approach, combining field trial data with collated global environmental data to evaluate the factors associated with yields for multiple SFAs side by side. Specifically, we gather results from numerous experiments worldwide that compared these practices (Fig. 1: agroforestry, cover cropping, no-tillage, and organic farming) against CM controls, and for each site, we overlay information on climate, soil, and topography drawn from global datasets. This approach allows us to assess how yield responses to different SFAs vary across a wide range of climatic zones, soil conditions, and landscape positions. By analyzing many practices and environmental variables together, our study provides a more comprehensive, comparative perspective on SFAs outcomes than previous analyses focused on a single practice. We aim to identify which combinations of practice and environment tend to produce positive yield results and where trade-offs might occur, thereby offering insights into the contexts in which SFAs can best contribute to both food security and sustainability.

## Materials and methods

A global dataset on SFAs was compiled, covering AF, CC, NT, and OF across major crop groups (maize, rice, soybean, wheat, etc.). Each observation was linked with climate, soil, and topographic variables. Yield effects were calculated as the percentage change in yield relative to conventional management, and moderator analyses were conducted across environmental categories. Statistical significance was assessed using 95% bootstrapped confidence intervals, while density plots and Jackknife resampling were applied to test for publication bias and robustness.

### Data collection

**Fig. 2:**
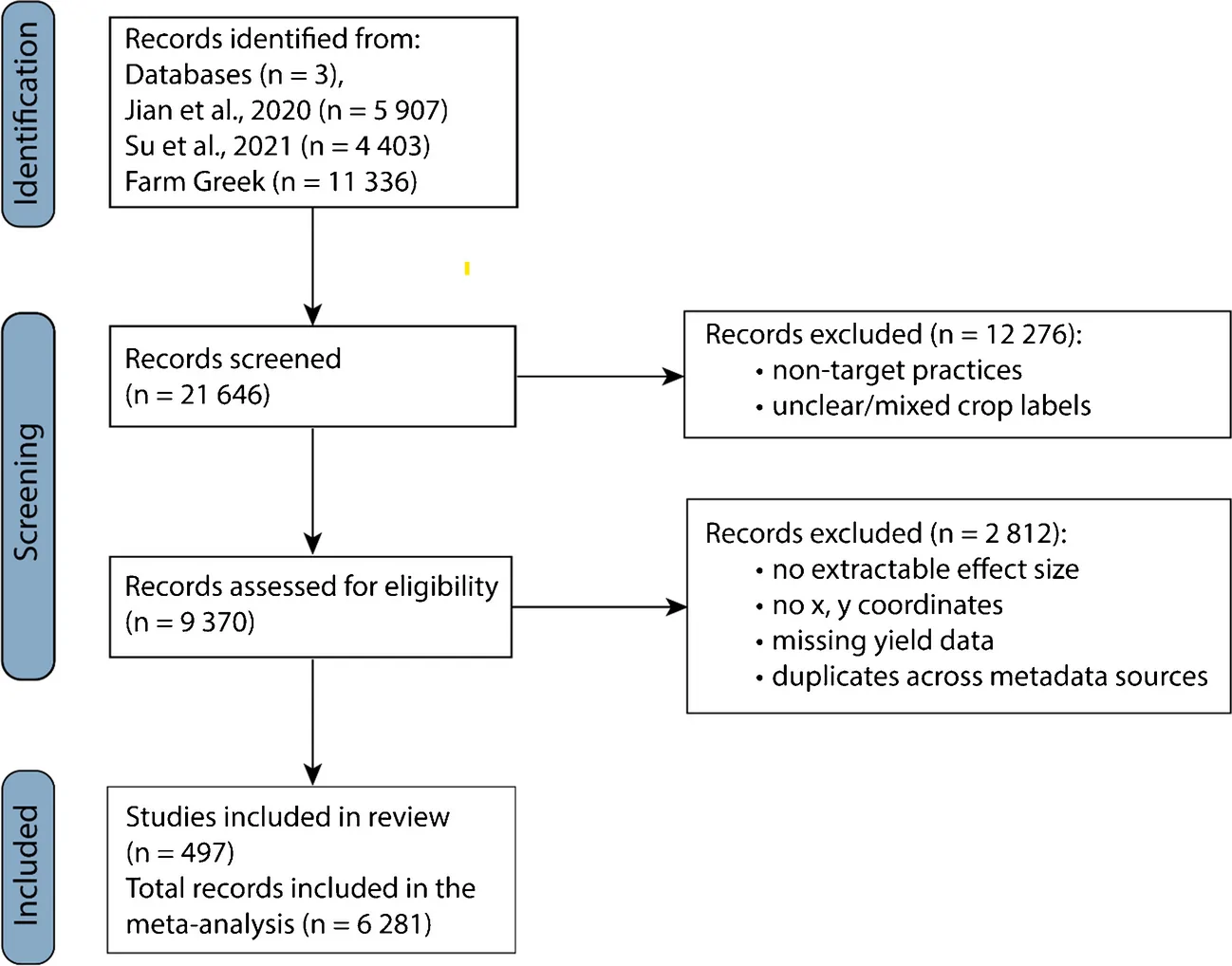
PRISMA-SR flow diagram, representing the stepwise process of record identification, de-duplication, and additional quality evaluation phase for the meta-analysis.

**Fig. 3:**
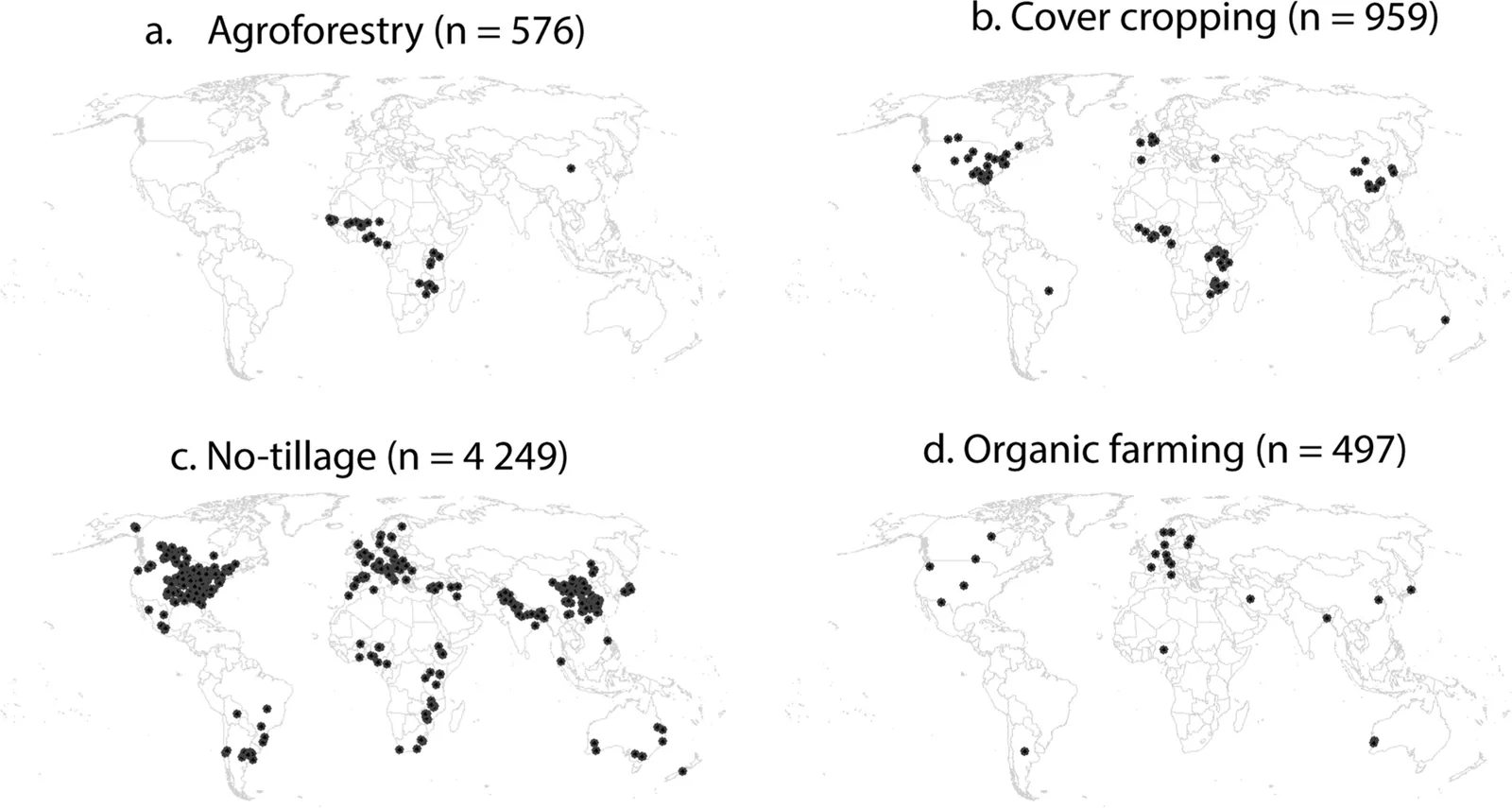
Global distribution of the study sites. **a** agroforestry; **b** cover cropping; **c** no-tillage; **d** organic farming.

Following PRISMA 2020 (Fig. 2) guidelines, we compiled datasets from published global meta-analyses Su et al. (2021), Jian et al. (2020), and the Farmgeek repository (https://www.farmgeek.xyz), yielding 21 646 records. The Farmgeek repository, considered as an additional source, is an open-access, community-driven platform that synthesizes peer-reviewed literature on agricultural management practices and food system interventions. It provides a harmonized, geospatially explicit database of paired yield observations drawn from field experiments conducted worldwide. During screening, records with unclear crop labels and non-target management categories were removed as well as those with no extractable yield data, missing data, no provision of coordinates, or duplicates across database and with extreme (>2.5) effect size (ES). In total, 497 studies met all eligibility criteria resulting in a total of 6 281 comparisons (Fig. 3) between SFAs and conventional management (CM).

After compiling the data, the crop types were classified into seven groups with the most cultivated crops in the world such as maize, wheat, soybean, and rice considered separately. The remaining crops were categorized cereal, cash-crop, and vegetable and fruits and others (see Table 1 in supplementary material). The compiled data cover the following SFAs: agroforestry (AF), cover cropping (CC), no-tillage (NT), and organic farming (OF). In line with Jian et al. (2020), we define the four SFAs as follows:

- Agroforestry (AF): trees or shrubs integrated in the same fields with crops and/or pastures, sometimes in combination with (grazing) livestock.
- Cover croppings (CC): crops planted in fallow periods, either in rotation or alongside the main crops.
- No-tillage (NT): cropping systems causing minimal or zero soil disturbance through tillage.
- Organic farming (OF): although the exact legal definition of OF varies across nations (Seufert et al. 2017), it relies on using organic fertilizer inputs instead of synthetic fertilizers, such as compost or green manure, and prohibits the application of pesticides.

We acknowledge that sustainable farming systems and practices frequently overlap in real-world applications; for example, cover croppings are often used as green manure within OF systems. However, in the present study, we distinguish organic farming from the CC category because OF explicitly prohibits the use of synthetic fertilizers and pesticides, whereas CC as defined above does not imply these restrictions. In addition, although AF and OF serve as comprehensive farming systems while NT and CC are more specific field-level practices, we adhere to methodologies established by prior meta-analyses (Jian et al. 2020), which treat them as comparable management categories for synthesis purposes. To enhance conceptual clarity, we collectively refer to these diverse approaches as Sustainable Farming Approaches (SFAs), encompassing both individual practices (e.g., NT, CC) and integrated farming systems (e.g., AF, OF).

### Environmental moderators

The impact of the SFAs on crop yields was conditioned on three environmental components: climate, soil properties, and topography. For each of the three components, several indicators were identified (Table 1).

- Climate variables: This study used two climate proxies, aridity and growing degree days (GDD), to characterize moisture and thermal conditions, respectively. The considered aridity index is defined as the ratio of mean annual precipitation to mean annual potential evapotranspiration. Consequently, it represents an annual climatic indicator rather than growing-season moisture availability. In regions with strongly seasonal climates, growing-season water availability may diverge from annual averages. Consequently, aridity was used here as a broad indicator of climatic dryness rather than a precise measure of within-season water stress. The global aridity index for the 1970–2000 period was obtained from the Consortium for Spatial Information (1 km) (Zomer et al. 2022). Growing degree days (GDD, ~1 km) were obtained from Ahvo et al. (2023) and represent cumulative heat availability derived from daily temperature data, calculated by summing temperatures above a base threshold (with an upper cut-off) and averaged over the period 1990–2010. While GDD is widely used as an indicator of thermal conditions relevant for crop development, in this study, it reflects general climatic heat availability rather than crop-specific growing-season accumulation. Growing-season GDD would provide a more crop-relevant measure; however, the databases considered in this study do not consistently report crop-specific planting and harvest dates at the site level. We therefore used standardized annual GDD as a consistent and reproducible indicator across all sites, noting that this approximation may be less accurate in regions with year-round growing conditions, where annual and growing season heat accumulation may diverge.
- Topographic variables: Elevation and slope were the topographic variables considered in this study. These data (1 km) were obtained via the platform provided by the global study of Amatulli et al. (2018). The landform grid data (~1 km) was sourced from the study of Iwahashi and Yamazaki (2022).
- Soil properties: Global soil properties such as soil texture (sand, silt, clay), bulk density (BD), soil organic carbon (SOC), pH, and soil types were downloaded from the SoilGrids (250 m) platform which is a global soil information system developed by ISRIC – World Soil Information (Poggio et al. 2021). The global stock of soil Olsen phosphorus (1 km) came from the global study carried out by McDowell et al. (2023).

**Table 1:** Environmental variables (in brackets are abbreviations).

Component	Indicators	Unit	Resolution
Climate	Growing degree days for maize (GDD-maize)	°C	0.083°
	Growing degree days for wheat (GDD-wheat)	°C	0.083°
	Growing degree days for rice (GDD-rice)	°C	0.083°
	Growing degree days for soybean (GDD-soybean)	°C	0.083°
	Aridity index (aridity)		0.083°
Soil properties	Soil texture	%	250 m
	pH		250 m
	soil organic carbon (SOC)	g kg⁻^1^	250 m
	Soil Olsen phosphorus concentrations (phosphorus)	mg kg⁻^1^	1000 m
	Bulk density (bd)	g cm⁻^3^	250 m
	Soil type		250 m
Topography	Slope	%	0.00083°
	Digital elevation model (dem)	m	0.00083°
	Geomorphological landform		0.00083°

To integrate the meta-analysis data with environmental variables, we overlaid spatial datasets of input factors such as climate, topographic and soil properties, available in raster format with the geographic coordinates of the SFAs reported in the meta-studies. Using these coordinates, we extracted corresponding environmental variable values for each observation point. This spatial extraction and data processing were carried out using R software, enabling the linkage of SFAs’ yield data with environmental conditions.

### Data analysis

The management effect was calculated as the percentage change in yield of the treatment relative to the conventional management control (ES= 100*((*X*_T_ − *X*_CM_)/*X*_CM_)) where *X*_T_ and *X*_CM_ are the yield value under treatment (AF, CC, NT, or OF) and control, respectively. A moderator analysis was conducted to determine the SFAs effects on the ES. This analysis was carried out by grouping the metadata into the following categories:

- Crop groups: The previously defined crop groups were considered: maize, wheat, soybean and rice, cereal, cash-crop, and vegetable and fruits and others.
- Bulk density (BD): Low BD values indicate permeable soils that facilitate root access to water and nutrients, whereas high BD values denote compacted soils with high mechanical impedance, limiting root growth. BD was classified into three categories: low (< 1.2 g cm⁻^3)^, moderate (1.2 g cm⁻^3^ ≤ BD < 1.47 g cm⁻^3^), and high (BD ≥ 1.47 g cm⁻^3^) (Chen and Weil 2011).
- pH: Three categories were considered: acidic (pH < 6.3), neutral (6.3 ≤ pH < 7.4), and alkaline (pH ≥ 7.4).
- Phosphorus: The P distribution classes were low (*P* < 10.9 mg kg⁻^1^), moderate (10.9 ≤ *P* < 21.4 mg kg⁻^1^), and high (*P* ≥ 21.4 mg kg⁻^1^) (Sims 2000).
- Soil organic carbon: Three categories were considered: low (SOC < 5 g kg⁻^1^), moderate (5 ≤ SOC < 10 g kg⁻^1^), and high (SOC ≥ 10 g kg⁻^1^). (Lefèvre et al. 2017)
- Soil texture: Soil texture was classified using the HYPRES (European) soil texture classification system (Wösten et al. 1999) implemented in the *soiltexture* R package (Moeys et al. 2023). The resulting texture classes were subsequently aggregated into three broad groups: fine, medium, and coarse.
- Soil types: Classes of soil types were used as defined on the SoilGrid platform (see Table 2 in supplementary material).
- Aridity was divided into five categories: hyper-arid (AI < 0.05), arid (0.05 *≤* AI < 0.2), semi-arid (0.2 *≤* AI < 0.5), sub-humid (0.5 *≤* AI < 0.65), and humid (AI ≥ 0.65) (Zomer et al. 2022).
- Growing degree days were classified into five intervals: <800, 800 ≤ GDD < 2700, 2700 ≤ GDD < 4000, 4000 ≤ GDD < 6000, and 6000 ≤ GDD ≤ 10,000 °C·year⁻^1^ (Parthasarathi et al. 2013).
- Elevation was classified into three categories: <250 m, 250 *≤* elevation *<* 1000 m, and ≥ 1000 m.
- Slope was classified into five categories: (Jahn et al. 2006): < 0.2%, 0.2 ≤ slope < 1%, 1 ≤ slope < 5%, 5 ≤ slope < 15%, and ≥ 15%.
- Landform: The initial 22 landform classes were reduced to 15 by grouping similar contour line classes (see Table 3 in supplementary material).

Variance information was unavailable for most primary studies, precluding the use of weighted or random-effects meta-analysis. We therefore estimated uncertainty around mean effect size using a nonparametric cluster bootstrap, which accounts for dependence among observations originating from the same study (Adams et al. 1997; Field and Welsh 2007). Each study was assigned a unique identifier constructed from the normalized reference, crop classification, publication year, and reported location coordinates. This ensured consistent clustering even when individual studies contributed multiple observations. We produced 1 000 bootstrap replicates by resampling study identifiers with replacement and recalculated the respective cluster-level means. This approach preserved within-study dependence while enabling between-study variation to naturally propagate throughout the bootstrap process. An ES was considered statistically significant when its confidence intervals excluded zero. Because this approach does not explicitly model between-study heterogeneity, results should be interpreted as descriptive and robust, consistent with recent applications of bootstrap-based uncertainty assessment in agricultural and environmental meta-analyses (Jian et al. 2020; Ren et al. 2023).

### Publication bias and sensitivity analysis

Given the limited reporting of variance or standard error estimates across primary studies, conventional tests for publication bias (e.g., Egger’s regression or funnel plots) could not be applied (Egger et al. 1997; Higgins et al. 2019). To address this limitation, we examined the distribution of ES using density plots as a qualitative proxy for funnel plots, assessing potential asymmetry that might indicate publication bias (Basche and DeLonge 2017; Joshi et al. 2023). Furthermore, we performed a Jackknife sensitivity analysis (Philibert et al. 2012) to test the robustness of the results. Each study was assigned a unique identifier, and data from one study were removed sequentially in each iteration to evaluate the influence of individual studies on the pooled mean of ES. As an additional exploratory check, we inspected potential small-study effects by plotting ES against study sample size to ensure that effect magnitude was not systematically related to study scale. Together, these analyses provided a qualitative yet comprehensive assessment of the robustness and potential bias in the meta-analytic results.

## Results and discussion

The large-scale implementation of SFAs necessitates a comprehensive understanding of the underlying processes and mechanisms influencing crop yield across diverse environmental contexts. Although existing studies have reported a range of outcomes, such as yield increases, decreases, or no significant change, many have not thoroughly examined the underlying biophysical factors that influence these yield responses (Giller et al. 2021; Ren et al. 2023). However, such knowledge is crucial for context-specific implementation of such practices. This study provides a comprehensive assessment of the impacts of different SFAs on crop yield, considering a broad range of crop groups, climate regimes, soil properties, and topographic characteristics.

### Crop yield change across practices

**Fig. 4:**
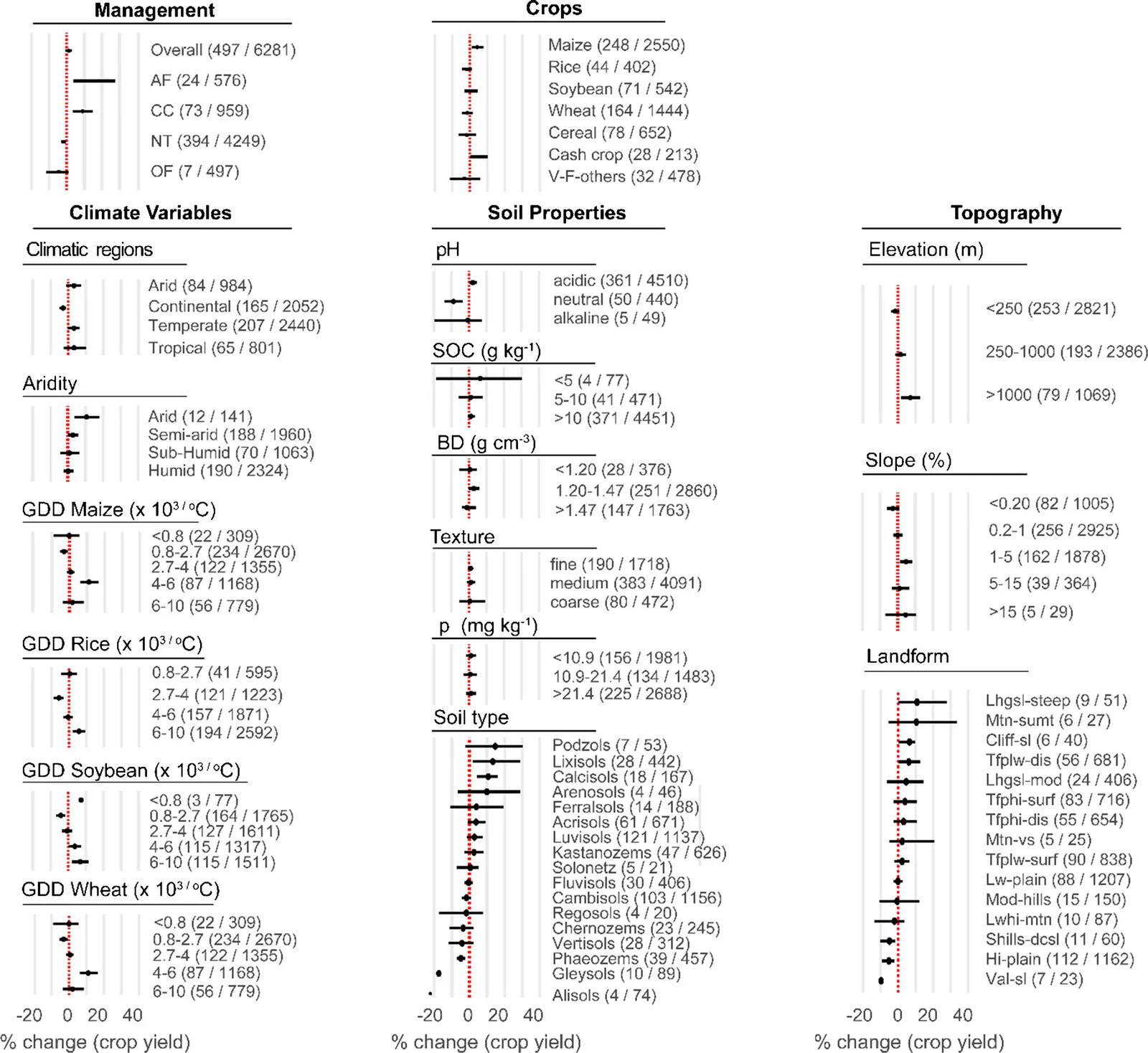
Distribution of the percentage change of the effect size between sustainable farming approaches, crop groups, soil properties, topography, and climatic variables. Points show means; error bars are 95% CIs. First/second number in bracket: number of studies/number of observations. Categories whose 95% CIs exclude 0 (vertical red line) differ significantly from controls. AF, agroforestry; CC, cover cropping; NT, no-tillage; OF, organic farming; V-F-others, vegetable, fruits, and others; P, phosphorus; BD, bulk density; GDD, growing degree days; SOC, soil organic carbon; Mtn-sumt, mountain summit; Cliff-sl, cliff slope; Lwhi-mtn, lower/hilly mountain; Shills-dcsl, steep hills/dissected cliff slope; Lhgsl-steep, large highland slope steep; Lhgsl-mod, large highland slope moderate; Mtn-vs, mountain valley slope; Mod-hills, moderate hills; Tfphi-dis, terrace/fan/plateau (high, dissected); Tfphi-surf, terrace/fan/plateau (high, surface); Val-sl, valley slope; Tfplw-dis, terrace/fan/plateau (low, dissected); Tfplw-surf, terrace/fan/plateau (low, surface); Hi-plain, high plain (sinks < 50%); Lw-plain, low plain (sinks < 50%).

Across the entire dataset, sustainable farming approaches (SFAs; Fig. 4) showed no significant overall effect on yield, with an average change of 1.1% (95% confidence interval, –0.7 to 3.0%). This small and non-significant mean effect has limited practical relevance, as it reflects the aggregation of practices and environments with widely divergent outcomes. Consequently, the key agronomic and policy insights lie in context-specific responses rather than in the global mean. Overall, these findings indicate that yields under SFAs were broadly comparable to those under conventional management. However, the magnitude and direction of yield responses varied substantially across individual practices and environmental conditions.

AF and CC were associated with significant yield increases of 14.5% (3.6–27.5%) and 8.9% (3.2–14.7%), respectively. While these findings align with previous research highlighting the beneficial effects of diversified cropping systems, the magnitude of the effect differs across studies (Peng et al. 2024; Ren et al. 2023; Tully and Ryals 2017). For example, Ren et al. (2023) recorded increased crop yield by 11% and 66% for CC and AF, respectively. In contrast, our observed increase for CC is substantially higher than the global average of 2.6% reported by Peng et al. (2024), yet slightly lower than the 9.2% increase reported in cases involving leguminous cover croppings, which is most likely due to their nitrogen-fixing abilities (Abdalla et al. 2019). These variations among studies are likely attributable to differences in soil conditions, climate, and management practices. In contrast, NT showed a small but statistically significant yield reduction (−1.8%, –3.4 to –0.2%) whereas OF showed no significant overall effect on yield (−4.4%, –11.7 to 1%), which may reflect challenges related to nutrient availability, weed pressure, or delayed adaptation of these systems in certain contexts (Mwangi et al. 2024; Ponisio et al. 2015).

### Crop yield change across climate types

Yield effects varied across climate zones and temperature regimes. Across climate zones (Fig. 4), SFAs showed no significant yield effect in arid (3.2%; 95% CI –1.1 to 7.1%), temperate (3.1%; 95% CI −0.1 to 6.5%), and tropical regions (3.1%; –2.6 to 10.1%), whereas a significant yield reduction was observed in continental regions (–3%; –5 to –1.1%). However, the patterns differed somewhat when using the aridity index classification, indicating sensitivity to how climate conditions are defined.

Yield effects were significantly positive under arid (aridity index class 0.05–0.20) conditions (10.5%; 3.6−18.2%) while no significant effect was observed in semi-arid (aridity index class 0.20–0.50) zones (2.8%; −0.1 to 5.8%). In sub-humid to humid environments (aridity index class > 0.50), yield responses were generally neutral, with confidence intervals overlapping zero. Such results indicate that SFAs are particularly advantageous under water-limited conditions, where improved soil structure, organic matter, and water retention enhance crop productivity and resilience.

**Fig. 5:**
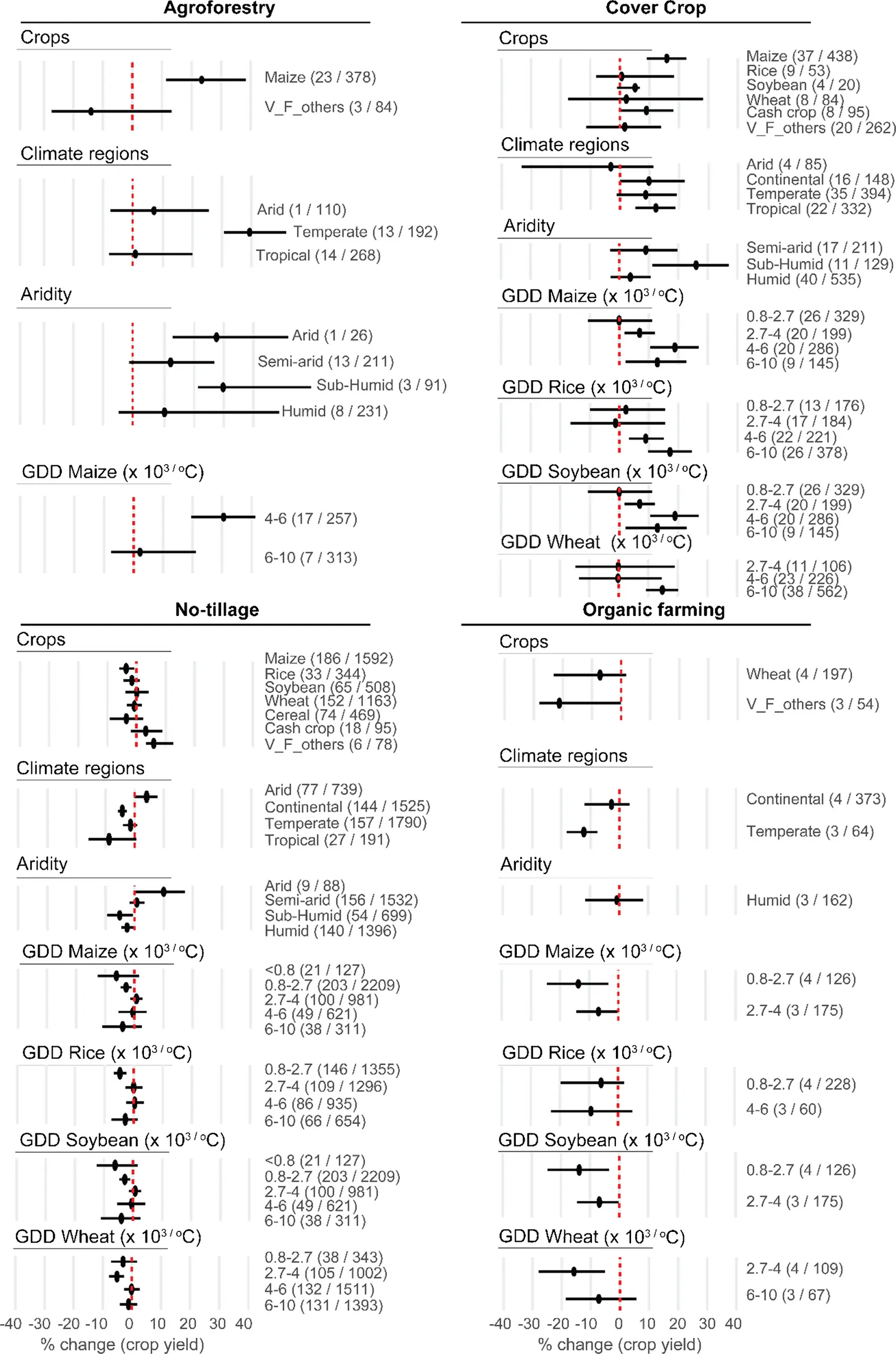
Distribution of effect size across crop groups and climate variables for different sustainable farming approaches. Points show means; error bars are 95% CIs. First/second number in bracket: number of studies/number of observations. Categories whose 95% CIs exclude 0 (vertical red line) differ significantly from controls. V-F-others, vegetable, fruits and others; GDD (× 10^3^, °C), growing degree days.

Among individual practices (Fig. 5), AF and CC both showed significant positive yield effects in sub-humid climates (AF: 29.8%, 21.3–59.6%; CC: 26.1%, 11.3–37.1%). AF additionally showed strong gains in temperate (39.4%; 30.7–51.8%) and arid (27.6%; 12.7–51.8%) conditions, while CC showed significant effects in tropical (12.1%; 5.2–18.6%) and continental (9.7%; 0.1–21.9%) climates. Effects of both practices were positive but not statistically significant in humid and semi-arid zones. NT, by contrast, showed neutral to negative effects across most climates, with statistically significant yield reductions in continental (−4.3%; −5.7 to −2.8%), sub-humid (−5.1%; −9.4 to −0.6%), and humid (−2.6%; −4.6 to −0.3%) conditions. In arid and semi-arid zones, NT showed positive but non-significant responses.

The relatively high effectiveness of AF in dry climates and the positive but non-significant trend for NT in arid and semi-arid zones likely reflect their ability to conserve soil moisture, reduce erosion, and maintain soil cover, which are essential mechanisms for protecting crops from drought stress. In contrast, CC can be constrained in arid climate regions, as cover croppings may compete with main crops for limited water, reducing overall yield (Teasdale and Mohler 2000). These findings underscore the value of AF in particular for stabilizing production in dry environments where conventional methods often exacerbate soil degradation and moisture loss. This further highlights the potential of AF to enhance resilience and productivity under increasingly dry conditions (Kuyah et al. 2019; Pittelkow et al. 2015), a finding that is especially relevant given the projected expansion of arid zones due to climate change (IPCC 2021).

In more humid climates, the picture shifts. AF and CC both showed significant positive effects in sub-humid conditions, while NT tended to reduce yields in sub-humid and humid zones, possibly reflecting waterlogging, disease pressure, or reduced nutrient availability under reduced tillage in wetter soils. The notable gains of AF in temperate climates likely reflect improved nutrient cycling, reduced erosion, and enhanced soil structure, which are particularly valuable in wetter systems that are susceptible to nutrient leaching and runoff. CC’s significant positive effects in tropical and continental climates further suggest that its benefits are more reliably expressed where water availability is less limiting. Overall, the observed climatic patterns highlight that the performance of SFAs depends strongly on local water balance and management context. Tailoring the choice of SFAs to prevailing moisture regimes can therefore maximize productivity, resilience, and sustainability under both current and future climate conditions.

### Crop yield change across specific crops and growing degree days

Yield responses to SFAs showed strong variation both among crop types and across thermal regimes, reflecting the interaction between crop physiology, management, and climatic conditions (Figures 4 and 5). Across all SFAs, yield responses varied substantially among crop types. Overall (Fig. 4), significant yield increases were observed for cash crop (5.8%; 0.5–10.3%) and maize (4.1%; 1.1–7.6%), while other crop groups showed no significant effects. Practice-wise (Fig. 5), CC generally showed positive mean responses across crop types, although not all effects were statistically significant (Fig. 5). Significant increases were recorded mainly for maize under AF and CC and for cash crops under CC and NT. These findings indicate that maize and cash crops benefited most from SFAs adoption, though the extent of yield improvement depended on the practice applied. The higher gains under CC and AF suggest that practices enhancing soil cover and organic inputs are more effective than disturbance-reducing measures alone. Such practices improve soil structure, water retention, and nutrient cycling, thereby creating favorable conditions for high-input crops that rely on sustained nutrient and moisture supply, especially for maize (Allam et al. 2023; Baier et al. 2023). In contrast, the variable performance under NT may reflect slower soil fertility buildup and residue-related constraints, which can limit yield benefits in the short term.

Yield responses across growing degree days (GDD, °C y⁻^1^) further revealed strong temperature-dependent patterns. Across all SFAs (Fig. 4), maize, rice, soybean, and wheat showed positive yield responses in specific GDD ranges; however, these patterns were not consistently statistically significant across all intervals. At the SFAs level (Fig. 5), AF and CC generally showed stronger positive responses under higher GDD conditions (> 4000 °C y⁻^1^), particularly for maize, while CC also showed positive responses for rice, soybean, and wheat at higher GDD levels. Nevertheless, these effects were not uniform across all crops and GDD classes. These results demonstrate that temperature regimes may influence the effectiveness of SFAs, with AF and CC performing best in warmer conditions. This is likely because AF and CC generally enhance soil water retention through improved drainage and deeper rooting systems, particularly in AF, and help protect bare soil from direct evaporation of limited precipitation. Additionally, by reducing soil sensitivity to erosion, they improve nutrient retention, making cash crops and maize more resilient during periods of drought and intense rainfall (Blanco‐Canqui and Ruis 2020; Krishnamurthy et al. 2019).

### Crop yield change across soil properties

**Fig. 6:**
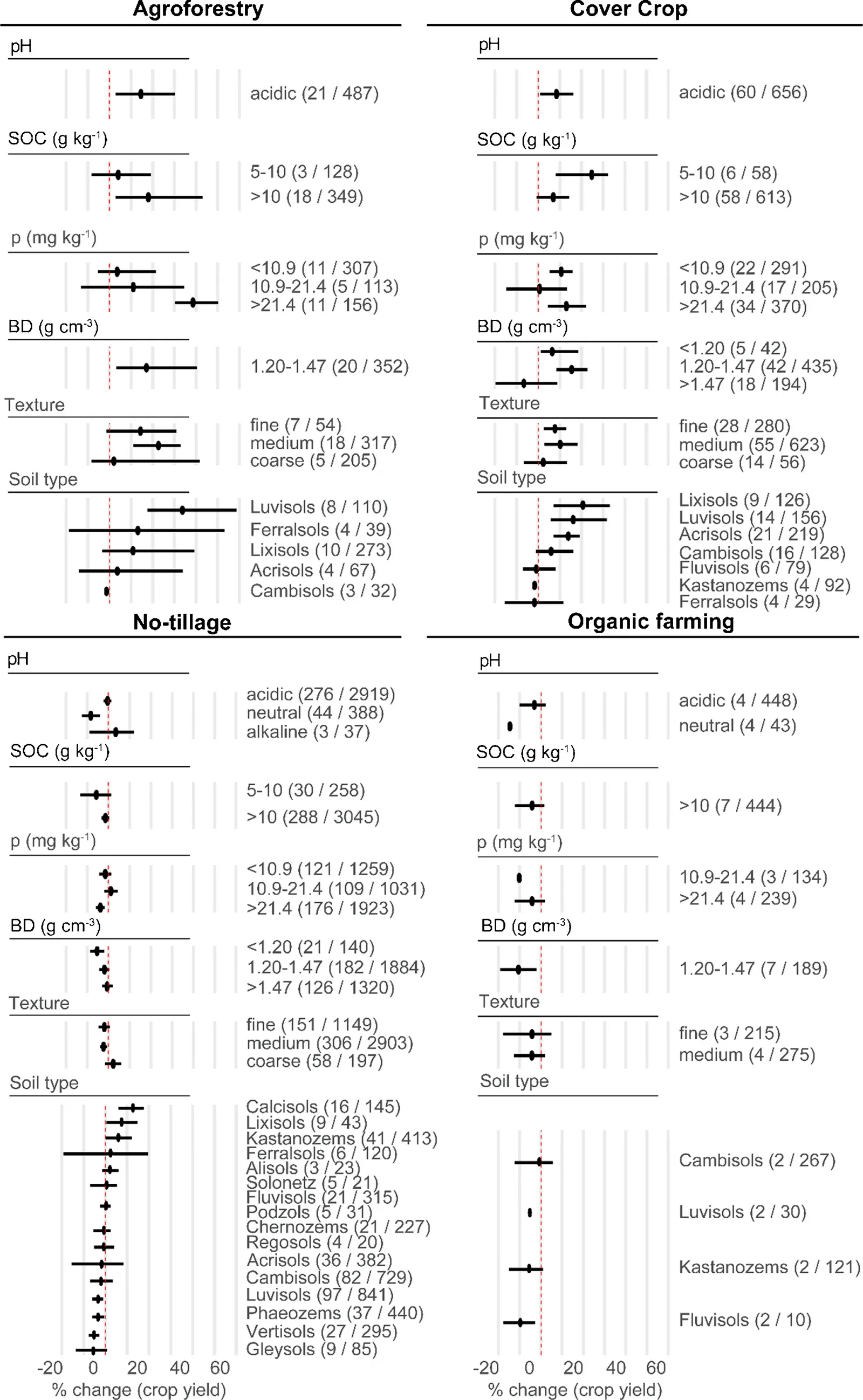
Distribution of effect size across soil properties for different sustainable farming approaches. Points show means; error bars are 95% CIs. First/second number in bracket: number of studies/number of observations. Categories whose 95% CIs exclude 0 (vertical red line) differ significantly from controls. P, phosphorus; SOC, soil organic carbon; BD, bulk density.

Yield responses to SFAs varied markedly across soil properties (Figures 4 and 6). When all SFAs were pooled (Fig. 4), positive yield responses were observed in low soil organic carbon (SOC < 5 g kg⁻^1^), coarse-textured, and acidic soils; however, these effects were not consistently statistically significant across all categories. Similarly, a trend of positive responses was observed in soils with low to moderate phosphorus levels (*P* < 21.4 mg kg⁻^1^) and low to medium bulk density (BD < 1.47 g cm⁻^3^), while neutral pH soils showed small or slightly negative responses.

Across individual SFAs (Fig. 6), CC showed more consistent positive responses with decreasing bulk density, although not all effects were statistically significant. Under higher SOC conditions (> 5 g kg⁻^1^), AF and CC showed smaller or non-significant responses, suggesting reduced marginal benefits in more fertile soils. These patterns suggest that SFAs may be more effective in nutrient-poor or structurally constrained soils, although results were not uniform across all soil categories. Enhancements in soil structure, nutrient cycling, and biological activity may contribute to these responses (Pittelkow et al. 2015; Vendig et al. 2023). Increasing phosphorus availability was generally associated with more positive yield responses, although these effects were not consistently statistically significant across all practices. Under NT, high P levels (> 21.4 mg kg⁻^1^) were associated with significant negative responses suggesting reduced relative benefits in already fertile systems.

In contrast, AF showed a significant yield increase under high P conditions (38%; 30.1–50%), while CC maintained positive but not consistently significant responses across P levels. This aligns with research showing that while AF systems enhance nutrient cycling, they still benefit from phosphorus supplementation, especially in P-deficient soils. Phosphorus is often a limiting nutrient in weathered tropical soils because it becomes fixed and unavailable to plants. However, its availability is crucial for both plant growth and biological nitrogen fixation, particularly in leguminous tree species commonly found in AF systems (Hu et al. 2025; Solangi et al. 2024). Studies have shown that P inputs can stimulate microbial activity, mycorrhizal associations, and root development, resulting in greater nutrient uptake and biomass production (Clausing and Polle 2020; Jansa et al. 2010). Thus, the 38% (30.1–50%) yield increase under high P in AF systems likely reflects the combined effects of improved nutrient acquisition, soil structure, and biological activity, supporting the idea that targeted P application in nutrient-poor soils can enhance the productivity of AF systems. While CC systems also sustained positive yield responses at high P concentrations (>21.4 mg kg⁻^1^), the magnitude of this response was comparable to that observed in low-P soils (<10.9 mg kg⁻^1^), indicating a plateau rather than a strong P-driven increase. This pattern suggests that CC might primarily improve P-use efficiency via enhanced rhizosphere activity, microbial P mineralisation, and mycorrhizal mobilization (Hallama et al. 2019) rather than relying on additional P inputs.

Soil texture and soil classification further influenced the magnitude of yield responses, although effects were not consistent across all categories. Overall (Fig. 4), no significant yield effects were observed across soil texture classes, including coarse-textured soils (0.6%; −5.5 to 9.1), medium-textured soils (1.2%; −1.1 to 3.4%), and fine-textured soils (1.0%; −1.5 to 3.9%). In contrast, significant yield increases were observed in Calcisols (10.7%; 4.2–16.1%) and Lixisols (13.3%; 1.9–28.8%), suggesting that these soil types may respond more positively to SFAs.

At the management level (Fig. 6), AF showed a significant yield increase in medium-textured soils (22.6%; 11–33%), which may reflect improved water retention and root development under these conditions. AF also showed strong positive effects in Luvisols (33.7%; 17.7–58.6%), indicating that soils with higher clay content and nutrient-holding capacity may benefit from enhanced biological activity and nutrient cycling. Under CC, significant yield increases were observed in medium- and fine-textured soils, as well as in Luvisols and Lixisols, suggesting that practices promoting soil cover and organic inputs may enhance soil structure and nutrient availability, particularly in more structured soils. In contrast, NT showed significant yield reductions in Gleysols and Phaeozems, which may be linked to drainage limitations and rapid organic matter turnover, respectively. These constraints can limit the effectiveness of reduced tillage in certain soil environments. Overall, these findings highlight that the effectiveness of SFAs depends strongly on soil characteristics, with more consistent benefits observed in specific soil groups such as Calcisols, Lixisols, and Luvisols, while responses in other soils remain variable and context-dependent.

### Crop yield change across topographic variables

**Fig. 7:**
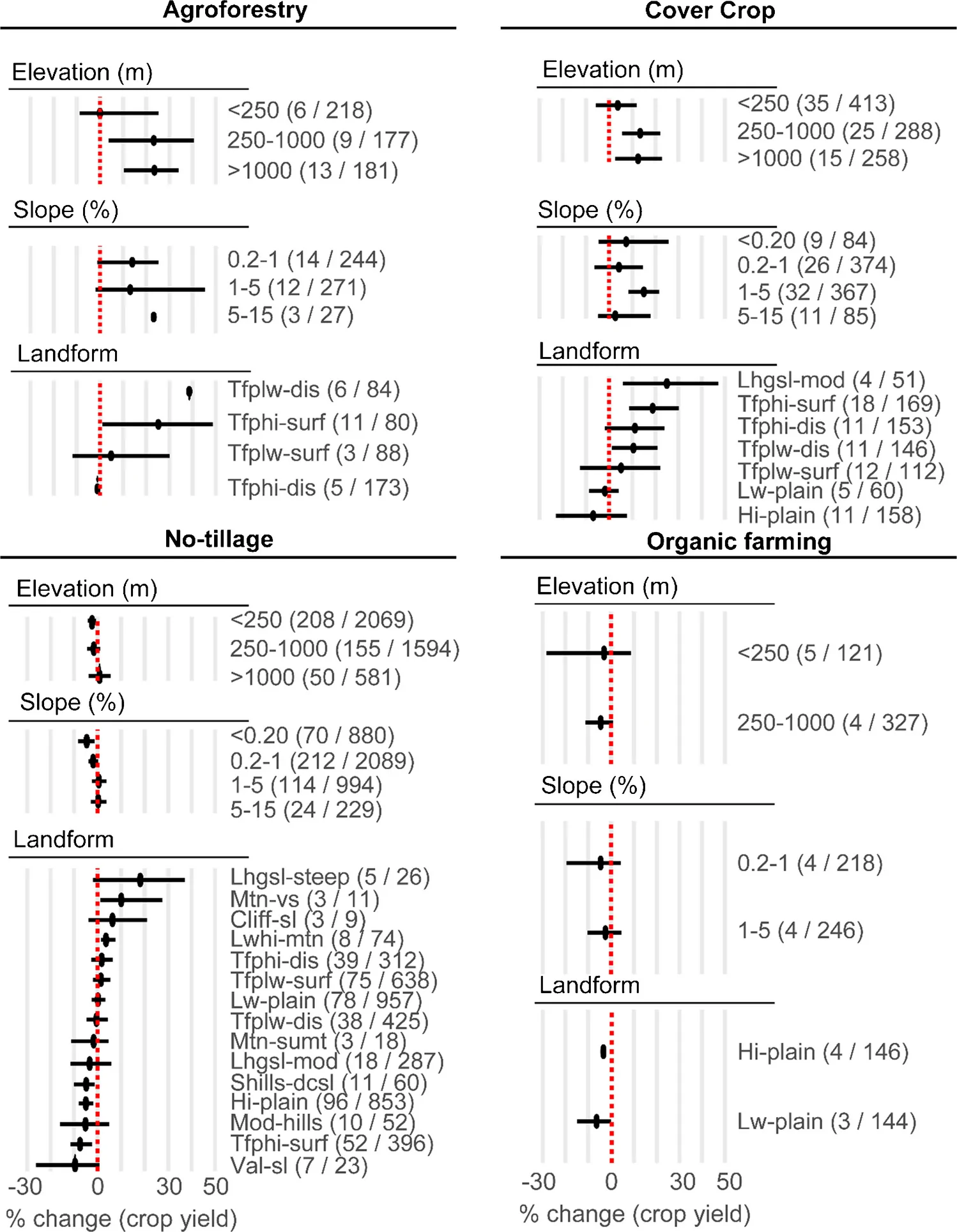
Distribution of effect size across topographic variables for different sustainable farming approaches. Points show means; error bars are 95% CIs. First/second number in bracket: number of studies/number of observations. Categories whose 95% CIs exclude 0 (vertical red line) differ significantly from controls. Lhgsl-steep, large highland slope steep; Mtn-vs, mountain valley slope; Cliff-sl, cliff slope; Lwhi-mtn, lower/hilly mountain; Tfphi-dis, terrace/fan/plateau (high, dissected); Tfplw-surf, terrace/fan/plateau (low, surface); Lw-plain, low plain (sinks < 50%); Tfplw-dis, terrace/fan/plateau (low, dissected); Mtn-sumt, mountain summit; Lhgsl-mod, large highland slope moderate; Shills-dcsl, steep hills/dissected cliff slope; Hi-plain, high plain (sinks < 50%); Mod-hills, moderate hills; Tfphi-surf, terrace/fan/plateau (high, surface); Val-sl, valley slope.

Yield responses to SFAs varied considerably with topographic conditions, including elevation, slope, and landform (Figures 4 and 7). When all SFAs were pooled (Fig. 4), significant yield increases were observed at elevations > 250 m, particularly in higher-elevation landforms such as mountain valley slopes and summits. Across landforms, yield responses were generally positive, although not all effects were statistically significant. Significant yield improvements were observed under gentle (1–5%) and strong slopes (15–30%), while responses in other slope classes were not consistently significant. These findings align with earlier studies demonstrating that yield responses to SFAs are strongly modulated by topographic gradients due to variations in soil moisture, erosion risk, and microclimate (Li et al. 2023; Nyairo 2024).

Across topographic gradients, AF showed significant yield increases in high-elevation areas (>250 m), with ES increasing from low to mid and high elevations. Significant positive responses were also observed in terrace and fan landforms (e.g., Tfplw-dis, Tfphi-surf), suggesting that AF performs well in relatively stable landscapes. These patterns likely reflect improved tree–crop interactions (Fahad et al. 2022) and enhanced drainage and erosion control in elevated or gently sloping landscapes (Ngaba et al. 2024), where deeper rooting and perennial cover increase soil moisture retention and reduce surface runoff.

CC also showed significant yield increases at mid- to high elevations (250–1000 and > 1000 m), particularly on gentle slopes (1–5%) and in moderately dissected landforms (e.g., Lhgsl-mod, Tfphi-surf, Tfplw-dis). These patterns suggest that CC may be especially effective in erosion-prone environments, where improved soil cover and aggregation can help protect the soil surface and enhance infiltration. This interpretation is supported by field evidence with Futerman et al. (2025) reporting a 29–58% reduction in rill erosion and substantial improvements in soil structure and infiltration under CC compared with bare soil.

NT responses were generally neutral to negative across most topographic classes. Significant yield reductions were observed on gentle slopes (0.2–1%), while most other slope, elevation, and landform categories showed no significant effects. A positive but statistically uncertain response was observed on locally steep hillslopes (Lhgsl-steep), suggesting that water retention on steeper terrain may partly offset limitations associated with reduced soil disturbance. These patterns suggest that NT performance is sensitive to local topographic context, though effects were not consistently significant across classes. For example, NT used in conjunction with terrace systems has been shown to reduce surface runoff by more than 90%, increasing soil moisture storage and buffering yields against both drought and intense rainfall events (Wang et al. 2023; Barbosa et al. 2025). Such mechanisms may partly explain the positive, if uncertain, response observed on locally steep hillslopes, though the limited evidence base for this class warrants cautious interpretation.

Overall, organic farming showed consistently neutral to negative yield responses across topographic settings, though none of the effects was statistically significant given the limited evidence base. Negative point estimates were observed at low and mid elevations, on gentle to moderate slopes, and in both high and low plain landforms. While these patterns tentatively suggest that OF may struggle to deliver yield benefits across varying topographic contexts, possibly due to nutrient constraints and drainage limitations (Tiwari 2021; Smith et al. 2020), the wide confidence intervals preclude firm conclusions, and more studies across diverse topographic settings are needed.

### Publication bias and sensitivity analysis

**Fig. 8:**
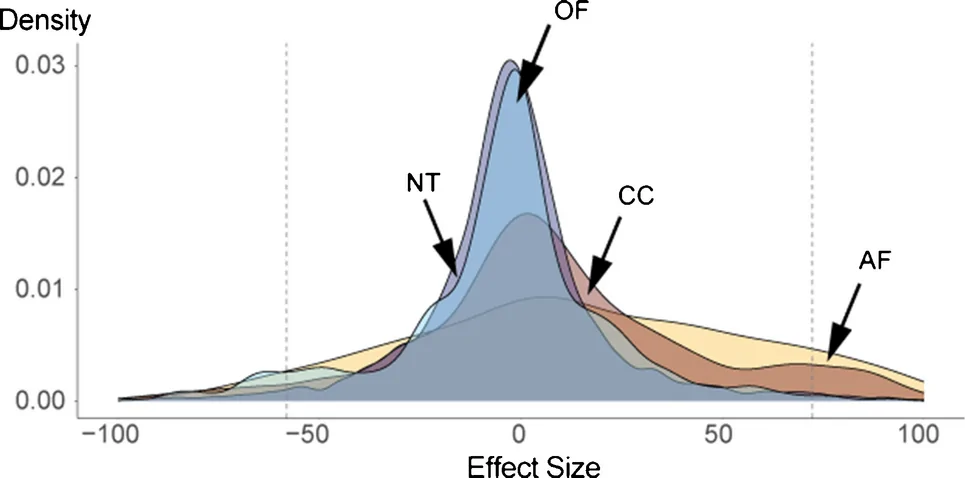
Density plots of effect size by farming approach. Dashed lines indicate 95% confidence interval bounds. AF, agroforestry; CC, cover cropping; NT, no-tillage; OF, organic farming.

**Fig. 9:**
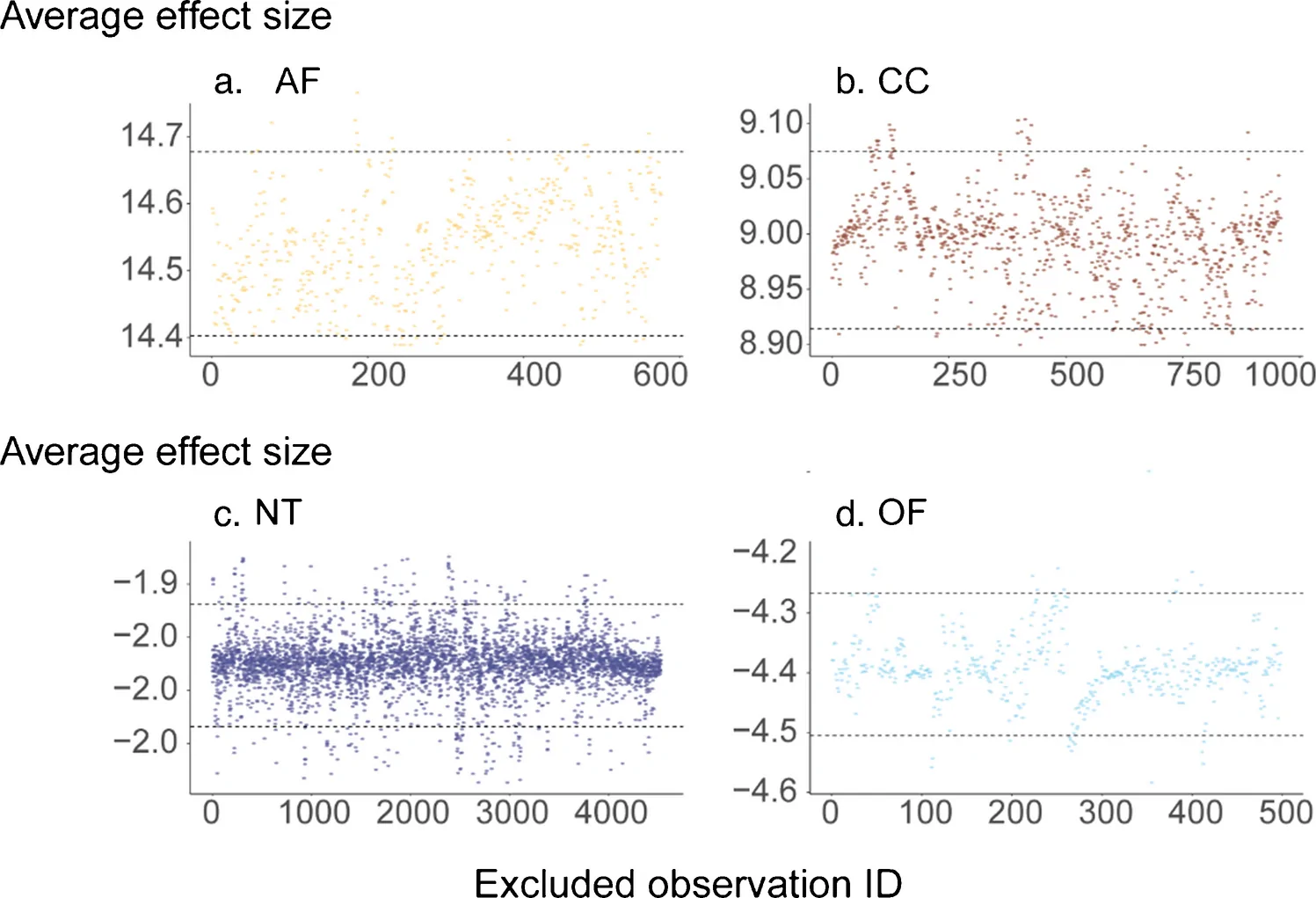
Leave-one-out sensitivity analysis of effect size estimates by farming approach. Panels show **a** agroforestry (AF), **b** cover cropping (CC), **c** no-tillage (NT), and **d** organic farming (OF). Dashed lines indicate 95% confidence interval bounds.

Publication bias and the robustness of the meta-analytic results were assessed using density plots and a Jackknife sensitivity analysis, complemented by an exploratory inspection of small-study effects. Density plots (Fig. 8) indicated that effect sizes were approximately normally distributed, with no evident asymmetry suggesting substantial publication or reporting bias. Similarly, the Jackknife sensitivity analysis showed that the exclusion of individual studies did not materially alter the pooled ES, as most recalculated estimates remained within the original 95% confidence interval (Fig. 9). Although a few exclusions produced estimates outside this range, their influence was minor given the large cumulative sample size and broad representation of experimental conditions, consistent with previous large-scale meta-analyses (Shackelford et al. 2019). Consequently, these influential studies were retained, as their exclusion would not meaningfully change the overall conclusions.

**Fig. 10:**
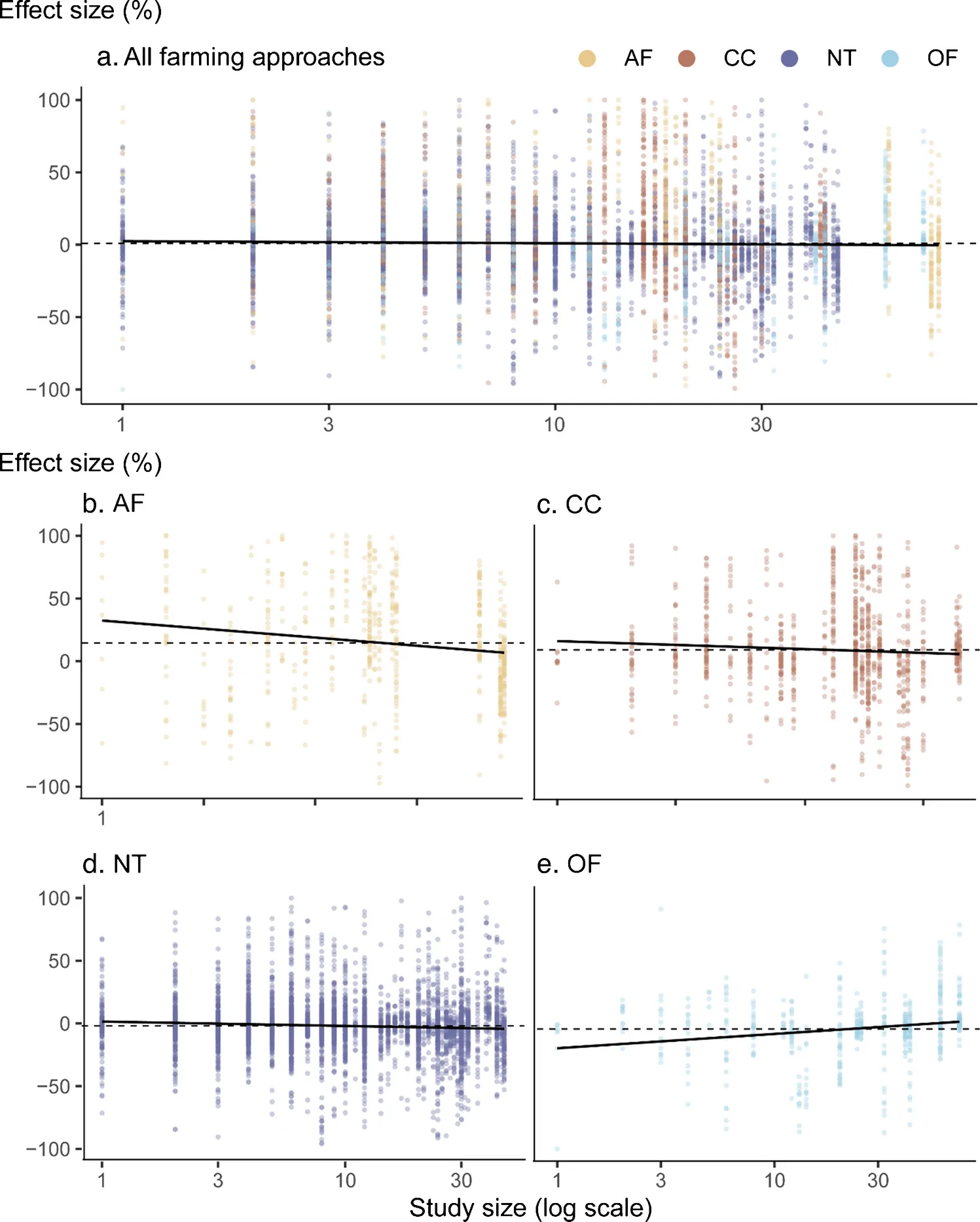
Relationship between effect size (%) and study size (log scale) across farming approaches. Panel **a** shows all farming approaches combined, while panels **b** agroforestry (AF), **c** cover cropping (CC), **d** no-tillage (NT), and **e** organic farming (OF) show the corresponding relationships for each farming approach individually. Points represent individual observations; solid lines indicate linear trends and dashed lines denote mean effect size.

The exploratory inspection of small-study effects (Fig. 10) showed no systematic relationship between ES and study size across all farming approaches combined (Fig. 10a), with the aggregate regression line remaining approximately flat around zero across the full range of study sizes. This supports the robustness of the overall pooled estimate. At the individual practice level, no meaningful trend was observed for NT (Fig. 10d), while OF (Fig. 10e) showed a slight positive slope, suggesting a marginal underestimation of effects in smaller studies. CC (Fig. 10c) displayed a weak negative trend, accompanied by substantial scatter and no clear directional pattern. In contrast, AF (Fig. 10b) exhibited a more pronounced negative relationship, with smaller studies tending to report larger positive effects, a pattern warranting cautious interpretation due to potential upward bias in the estimated magnitude. Nevertheless, the direction of the AF effect remained consistently positive across study sizes. Taken together, the absence of a systematic trend at the aggregate level, combined with inconsistent directions across practices, indicates that small-study bias does not materially compromise the overall meta-analytic conclusions, although the magnitude of the agroforestry (AF) effect may be overestimated and should therefore be interpreted with caution.

### Limitations of the study and future directions

The results of this study present the potential of SFAs to influence crop productivity with the variability in yield responses suggesting that context-specific adaptation is crucial. Policymakers and practitioners should consider local soil and climate conditions, as well as crop types, when promoting specific SFAs.

The number and spatial distribution of the studies included in this analysis may limit the generalizability of the results to underrepresented regions. The geographic coverage of NT and OF studies was heavily skewed toward Europe and North America (Fig. 3), while no AF studies were available from Latin America despite the region hosting extensive agroforestry systems covering hundreds of millions of hectares globally (Nair et al. 2022). Moreover, the dataset was dominated by NT observations compared to the other sustainable farming approaches (SFAs), which may have exerted a stronger influence on the pooled results. Although disaggregating results by practice helped reveal individual patterns, these imbalances highlight the need for more globally representative data, particularly for AF, CC, and OF.

Another limitation concerns the environmental data used in this analysis. Soil, climate, and topographic variables were obtained from global geospatial datasets rather than measured directly in the field. Although these datasets offer standardized global coverage, they introduce uncertainty due to coordinate inaccuracies and spatial mismatches between raster data and plot-level observations (Groß et al. 2023; Rohmer et al. 2024). Many meta-analyses report site locations only as approximate centroids, creating potential positional errors when linking field data to environmental covariates (Kinnee et al. 2020). An analysis of coordinate precision across all study sites (Supplementary Figure 1) indicates that 88.8% of records correspond to spatial uncertainties of ±550 m or greater (≤2 decimal places). The majority of locations (71.3%) are associated with two decimal places (≈ ±550 m), while 17.5% correspond to one decimal place or less (≈ ±5.5–55 km). A smaller subset of records (≈11%) exhibit higher nominal precision (≥3 decimal places), likely reflecting coordinate rounding or processing rather than true positional accuracy. Overall, recorded uncertainty is comparable to the resolution of climate and topographic datasets (~1 km) and exceeds the resolution of the SoilGrids dataset (250 m). Environmental values were therefore extracted using the nearest grid cell to the reported coordinate, consistent with standard practice in global meta-analyses (Joshi et al. 2023; Peng et al. 2024).

However, at the resolution of global datasets such as SoilGrids (250 m), this coordinate imprecision may result in the extracted environmental value representing a nearby location rather than the exact experimental plot, particularly in spatially heterogeneous landscapes. Such misassignment is expected to introduce random rather than systematic error, attenuating rather than inflating covariate effect sizes (Gurevitch et al. 2018; Carroll et al. 2006). We therefore used point extraction (nearest grid cell) as a consistent approach across datasets, recognizing that spatial averaging across multiple cells could introduce smoothing across distinct environments that may not reflect actual site conditions (Hengl et al. 2017; Poggio et al. 2021).

Differences in spatial resolution (e.g., SoilGrids (250 m) and CHELSA (1 km)) can also generate modifiable areal unit problems (MAUP), where averaged values fail to capture local variability (Maynard et al. 2023). Soil properties such as SOC, pH, and bulk density vary substantially within fields, and modelled data cannot fully reflect this fine-scale heterogeneity (Wu et al. 2023). Likewise, interpolated climate and DEM-based terrain attributes may obscure microclimatic differences that strongly influence yields (Dritsas and Trigka 2025). These uncertainties may affect the precision of detected environmental relationships. Future studies could reduce them by testing sensitivity to extraction scale or by integrating global rasters with field-measured variables where possible.

In addition, yield was the sole outcome metric considered in this study, despite the multifunctional goals of SFAs including carbon sequestration, biodiversity enhancement, and climate resilience. This narrow focus may miss important trade-offs and co-benefits that could influence adoption decisions by policy makers, land managers and farmers. The attribution of yield effects to individual SFAs is further complicated by the frequent bundling of multiple practices within the same study, especially in NT systems. For instance, AF can include alley cropping, forest farming, silvopastoralism, or riparian forest buffers (Nair et al. 2022; Feliciano et al. 2018; Sprenkle-Hyppolite et al. 2024), while CC species differ in their root structure with fibrous species such as ryegrass or oats controlling erosion more effectively than thick-rooted types like white mustard or fodder radish (Sharma et al. 2018). Such management diversity can obscure the individual contribution of each practice, highlighting the need for more detailed reporting and standardized classification in future analyses.

This study did not assess how different SFAs contribute to the resilience of farming systems in the face of increasingly frequent climate extremes, such as droughts and floods, which represents a critical dimension of food security in a changing climate. SFAs might have further potential to buffer yield losses during extreme events but could also bolster farmers’ (economic) resilience by reducing the risk of total crop failure (Raheem et al. 2025). Understandably, a modest, stable harvest achieved through enhanced soil health and water management may be preferable to a higher but highly variable yield that collapses under stress. By prioritizing resilience, future studies can explore how SFAs affect long-term yield stability, soil health, and ecosystem services under forecasted climate conditions along with how these advantages translate into more secure and sustainable livelihoods for farmers.

## Conclusion

This study provides the first comparative global assessment of how climate, soil, and topographic conditions shape yield responses to agroforestry, cover cropping, no-tillage, and organic farming within a common framework of biophysical moderators. The results demonstrate that yield responses to these sustainable farming approaches are not uniform but are strongly shaped by environmental context. Agroforestry and cover cropping showed statistically significant positive yield effects under specific aridity conditions, while no-tillage was consistently associated with statistically significant yield reductions in wetter environments, and organic farming showed no significant overall effect. Across soil and topographic gradients, yield responses were similarly heterogeneous, with significant effects confined to particular soil types and landscape positions.

These findings underscore that environmental heterogeneity, rather than the farming approaches themselves, largely governs yield outcomes. As a result, broad generalizations about the yield performance of sustainable farming approaches are unlikely to hold across diverse agroecosystems. Targeting practices to the conditions under which they are most effective will be essential for designing sustainable agricultural strategies that support both productivity and ecosystem resilience. Effective policy should therefore prioritize targeted incentives, regionally tailored guidance, and strengthened extension services to help land managers implement the practices best suited to their environmental conditions.

## Supplementary information

Below is the link to the electronic supplementary material.

ESM 1 (DOCX 84.9 KB)

ESM 2 (DOCX 21.7 KB)

## Data Availability

Topography data were obtained from https://www.earthenv.org/topography. The geomorphological landform grid data were obtained from https://gisstar.gsi.go.jp/terrain2021/. The global stock of soil Olsen phosphorus was accessed via the following platform. Data related to soil properties were downloaded from https://soilgrids.org/. The global aridity index was obtained from https://www.global-ai-pet.org/global-aridity-index-pet-database. The data generated and analyzed during this study, including the complete list of references selected for the meta-analysis, are available through the following Figshare repository: https://doi.org/10.6084/m9.figshare.32647386. Analytical scripts and supporting materials required to reproduce the analyses are publicly available at https://github.com/oziashounkpatin/Global-yield-variability-drivers.

## Code availability

All data analyses were conducted in R (version 4.2.2). The scripts used in this study are openly available on Github: https://github.com/oziashounkpatin/Global-yield-variability-drivers.

## Declarations

### References used for the meta-analysis

The complete list of the 497 references selected for the meta-analysis is available via a Figshare repository accessible through the following permanent link: 10.6084/m9.figshare.32647386.
