# Supplementary material for: Global determinants of yield variability under sustainable farming approaches across climate, soil, and topography. A meta-analysis
Source: Agron Sustain Dev. 2026 Jul 22;46(4):58. doi: 10.1007/s13593-026-01133-7 (PMC13391773; doi:10.1007/s13593-026-01133-7)

Supplementary Figure 1: Distribution of coordinate precision for study locations. Bars show the number of records by the number of reported decimal places (dp). One decimal place corresponds to an approximate spatial uncertainty of ±11 km, and two decimal places to ±1.1 km at the equator.


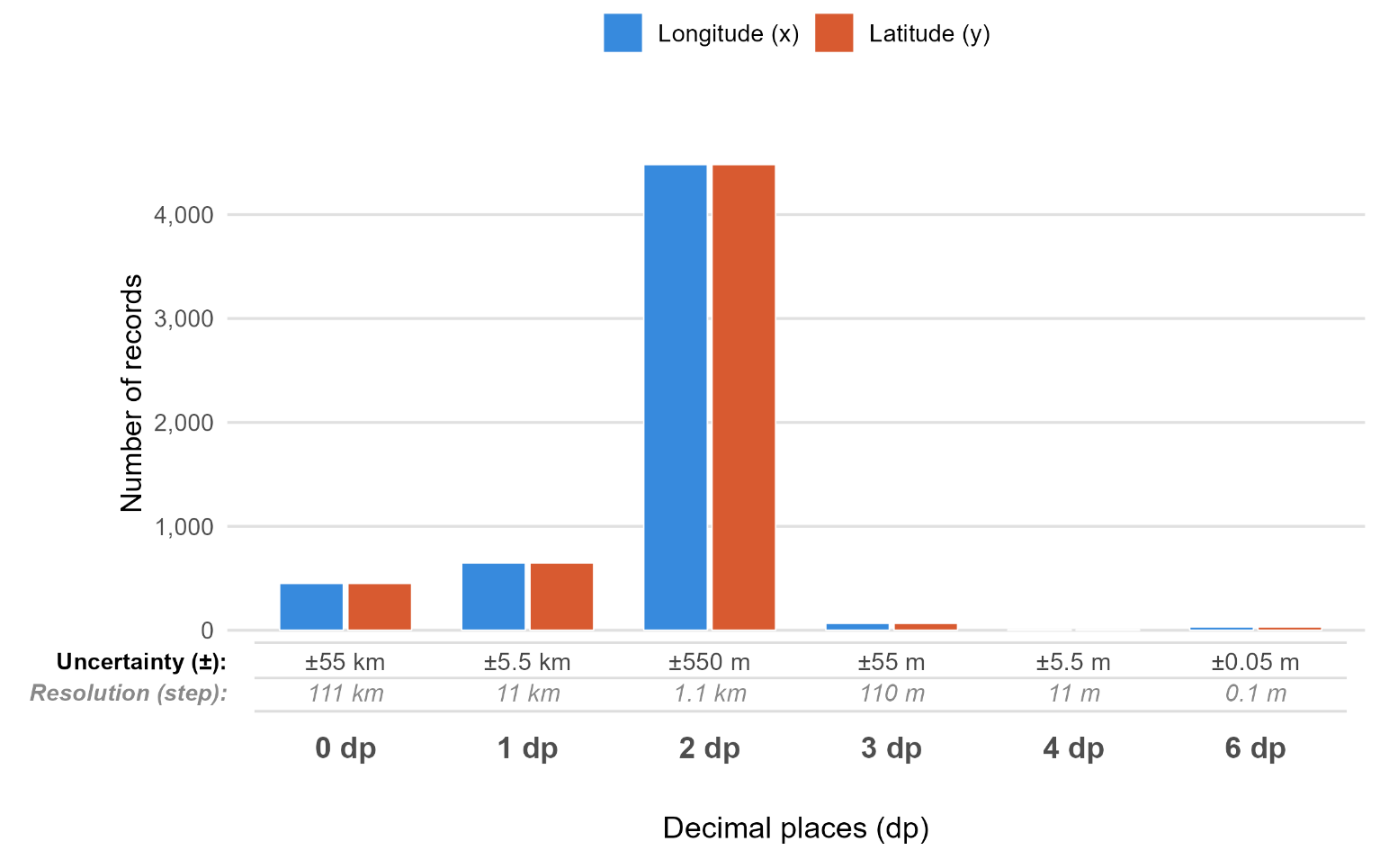

Supplement: Supplementary file 1 — (DOCX 84.9 KB) [file 13593_2026_1133_MOESM1_ESM.docx]
