# Supplementary material for: Global determinants of yield variability under sustainable farming approaches across climate, soil, and topography. A meta-analysis
Source: Agron Sustain Dev. 2026 Jul 22;46(4):58. doi: 10.1007/s13593-026-01133-7 (PMC13391773; doi:10.1007/s13593-026-01133-7)
Supplement: Supplementary file 2 — (DOCX 21.7 KB) [file 13593_2026_1133_MOESM2_ESM.docx]

**S**upplementary Table 1: Crop groups

| Crop | Crop group | Crop | Crop group | Crop | Crop  group |
| --- | --- | --- | --- | --- | --- |
| Corn | Maize | Cassava | V_F_others | Onion | V_F_others |
| Maize | Maize | Cauliflower | V_F_others | Pea | V_F_others |
| Sweet corn | Maize | Celery | V_F_others | Peach | V_F_others |
| Durum wheat | Wheat | Chickpea | V_F_others | Pepper | V_F_others |
| Spelt wheat | Wheat | Chilli | V_F_others | Physic nut | V_F_others |
| Wheat | Wheat | Cucumber | V_F_others | Pigeon pea | V_F_others |
| Rice | Rice | Choy sum | V_F_others | Pigweed | V_F_others |
| Soybean | Soybean | Citrus | V_F_others | Safflower | V_F_others |
| Barley | Other cereal | Clover | V_F_others | Satsuma mandarin | V_F_others |
| buckwheat | Other cereal | Cocoyam | V_F_others | Sesame | V_F_others |
| Millet | Other cereal | Coriander | V_F_others | Spinach | V_F_others |
| millet, finger | Other cereal | Cowpea | V_F_others | Squash | V_F_others |
| Oat | Other cereal | Dandelion | V_F_others | Strawberry | V_F_others |
| Pearl millet | Other cereal | Dill | V_F_others | Sugar beet | V_F_others |
| Rye | Other cereal | Eggplant | V_F_others | Sugarcane | V_F_others |
| Sorghum | Other cereal | Endive | V_F_others | Sunflower | V_F_others |
| Tef | Other cereal | Fennel | V_F_others | Sweet pepper | V_F_others |
| Triticale | Other cereal | Fenugreek | V_F_others | Sweet potato | V_F_others |
| Coffee | Cash crop | Fig | V_F_others | Potato | V_F_others |
| Cotton | Cash crop | Flax | V_F_others | Pulses | V_F_others |
| Jute | Cash crop | Garlic | V_F_others | pumpkin | V_F_others |
| Peanut | Cash crop | Grape | V_F_others | Taro | V_F_others |
| Tobacco | Cash crop | Green bean | V_F_others | Tomato | V_F_others |
| African eggplant | V_F_others | Hazelnut | V_F_others | Turmeric | V_F_others |
| Alfalfa | V_F_others | Japanese spinach | V_F_others | Turnip | V_F_others |
| Apple | V_F_others | Kidney bean | V_F_others | Vetch | V_F_others |
| Apricot | V_F_others | Kiwifruit | V_F_others | Vineyard | V_F_others |
| Banana | V_F_others | Lentil | V_F_others | Watermelon | V_F_others |
| Bauhinia trees | V_F_others | Lettuce | V_F_others | Yam | V_F_others |
| Bean | V_F_others | Linseed | V_F_others | Zucchini | V_F_others |
| Beet | V_F_others | Lupin | V_F_others | Quinoa | V_F_others |
| Black gram | V_F_others | Melon | V_F_others | Radish | V_F_others |
| Broad bean | V_F_others | Mung bean | V_F_others | Rapeseed | V_F_others |
| Broccoli | V_F_others | Mustard | V_F_others | Ribwort plantain | V_F_others |
| Cabbage | V_F_others | Oil palm | V_F_others | Runner bean | V_F_others |
| Carrot | V_F_others | okra | V_F_others |  |  |
|  |  |  |  |  |  |

Supplementary Table 2: Soil type classes

| Code | Soil class | Code | Soil class |
| --- | --- | --- | --- |
| 0 | Acrisols | 15 | Kastanozems |
| 1 | Albeluvisols | 16 | Leptosols |
| 2 | Alisols | 17 | Lixisols |
| 3 | Andosols | 18 | Luvisols |
| 4 | Arenosols | 19 | Nitisols |
| 5 | Calcisols | 20 | Phaeozems |
| 6 | Cambisols | 21 | Planosols |
| 7 | Chernozems | 22 | Plinthosols |
| 8 | Cryosols | 23 | Podzols |
| 9 | Durisols | 24 | Regosols |
| 10 | Ferralsols | 25 | Solonchaks |
| 11 | Fluvisols | 26 | Solonetz |
| 12 | Gleysols | 27 | Stagnosols |
| 13 | Gypsisols | 28 | Umbrisols |
| 14 | Histosols | 29 | Vertisols |

Supplementary Table 3: Landform classes

| Landform classes | Abbreviation |
| --- | --- |
| Mountain summit | Mtn_sumt |
| Cliff slope | Cliff_sl |
| Lower/hilly mountain | Lwhi_mtn |
| Steep hills / dissected cliff slope | Shills_dcsl |
| Large highland slope steep | Lhgsl_steep |
| Large highland slope moderate | Lhgsl_mod |
| Mountain valley slope | Mtn_vs |
| Moderate hills | Mod_hills |
| Terrace/fan/plateau (high, dissected) | Tfphi_dis |
| Terrace/fan/plateau (high, surface) | Tfphi_surf |
| Valley slope | Val_sl |
| Terrace/fan/plateau (low, dissected) | Tfplw_dis |
| Terrace/fan/plateau (low, surface) | Tfplw_surf |
| High plain (Sinks < 50%) | Hi_plain |
| Low plain (Sinks < 50%) | Lw_plain |
